# Sphingosine-1-Phosphate Receptor Subtype 1 (S1P1) Modulator IMMH001 Regulates Adjuvant- and Collagen-Induced Arthritis

**DOI:** 10.3389/fphar.2019.01085

**Published:** 2019-09-19

**Authors:** Jing Jin, Ming Ji, Rong Fu, Mingjin Wang, Nina Xue, Qiong Xiao, Jingpin Hu, Xiaojian Wang, Fangfang Lai, Dali Yin, Xiaoguang Chen

**Affiliations:** ^1^State Key Laboratory of Bioactive Substances and Functions of Natural Medicines, Institute of Materia Medica, Chinese Academy of Medical Sciences and Peking Union Medical College, Beijing, China; ^2^Beijing Key Laboratory of Non-Clinical Drug Metabolism and PK/PD Study, Chinese Academy of Medical Sciences and Peking Union Medical College, Beijing, China

**Keywords:** S1P1, S1P1 modulator, lymphocyte homing, rheumatoid arthritis, animal model

## Abstract

Sphingosine-1-phosphate receptor subtype 1 (S1P_1_) is essential for lymphocyte egress from lymphoid organs into systemic circulation and provides a well-defined drug target for autoimmune disorders. IMMH001, also called SYL930, is a specific S1P_1_/S1P_4_/S1P_5_ modulator. Here, we investigated the potential therapeutic effect of IMMH001 on rheumatoid arthritis (RA). IMMH001 rendered periphery blood lymphocytes insensitive to the egress signal from secondary lymphoid organs. Importantly, in both rat adjuvant-induced arthritis and collagen-induced arthritis models, IMMH001 treatment significantly inhibited the progression of RA and RA-associated histological changes in the joints of Sprague-Dawley rats, including hind paw swelling and arthritic index, and thus reduced the pathological score. Furthermore, IMMH001 markedly decreased proinflammatory cytokine and chemokine release from the damaged joints. These data demonstrated that IMMH001 is a promising drug candidate for RA treatment.

## Introduction

Rheumatoid arthritis (RA) is a severe lifelong autoimmune disease of the synovial joint tissue, characterized by chronic synovial inflammatory cell infiltration causing cartilage degradation and joint destruction (Oliver and Silman, 2006; Helmick et al., 2008). RA treatment aims to control pain and inflammation, reduce joint damage and disability, and maintain or improve physical function and quality of life (Ferro et al., 2017). Although the etiology of RA remains unclear, the disease has poor prognosis and limited treatment options (Scott et al., 1987; Yi et al., 2009). Patients are usually administered long-term disease-modifying anti-rheumatic drugs, such as methotrexate (MTX) (Yap et al., 2018). Many immune cells, such as T cells, B cells, and macrophages, play crucial roles in the pathogenesis of RA *via* stimulating proinflammatory cytokine and chemokine release (Silverman and Carson, 2003; Drexler et al., 2008; Iwamoto et al., 2008; Wang et al., 2011; Mellado et al., 2015; Sun et al., 2018; Wehr et al., 2018; Yap et al., 2018); therefore, targeting immune cells or proinflammatory cytokines is beneficial for RA patients (Asquith et al., 2015; Bullock et al., 2018; Littlejohn and Monrad, 2018; Mahajan and Mikuls, 2018; Zhao et al., 2018).

Sphingosine-1-phosphate (S1P) is a bioactive signaling molecule that regulates cell proliferation, migration, and cell–cell communications (Sadahira et al., 1992; Van Brocklyn et al., 2002). S1P binds to the G protein-coupled S1P receptor subtypes sphingosine-1-phosphate receptor subtype 1–5 (S1P_1–5_), which trigger a broad range of biological functions, including angiogenesis, endothelial barrier enhancement, blood vessel constriction, heart rate modulation, and lymphocyte trafficking (Spiegel and Milstien, 2002; Rosen et al., 2013). The immunomodulatory drug, FTY720 (fingolimod), which has been approved for the treatment of relapsing–remitting multiple sclerosis (Chiba, 2005; Brinkmann et al., 2010), is a S1P_1/3/4/5_ modulator with anti-inflammatory properties that regulates S1P_1_ degradation. The most serious adverse effects of FTY720 are bradycardia and atrioventricular block, which are caused by the non-selective binding of FTY720 to other subtypes of S1P receptors, particularly S1P_3_ (Sanna et al., 2004).

IMMH001, also called SYL930, was developed as a novel S1P_1_ agonist and has therapeutic effects on autoimmune encephalitis and psoriasis (Jin et al., 2014; Ji et al., 2018). To explore the detailed mechanism of IMMH001 and expand its indications, we analyzed the therapeutic effect of IMMH001 on RA by using rat adjuvant-induced arthritis (AA) and collagen-induced arthritis (CIA) models.

## Methods

### Cell Lines

hS1P_1_-CHO cells transfected with Chinese hamster ovary-K1 (CHO-K1) Gαqi5 with high S1P_1_ expression were purchased from the Multispan Inc. (Hayward, CA, USA). The cells were cultured in DMED/F12 medium supplemented with 10 μg/mL puromycin, 250 μg/mL hygromycin, and 10% fetal bovine serum.

### Animals

Male F344 rats (body weights of 160 to 180 g) or 6- to 8-week-old male Sprague-Dawley rats were obtained from the Vital River Laboratory Animal Technology Co., Ltd. (Beijing, China). All the rats were housed under standardized light- and climate-controlled conditions with free access to food and water. This study was carried out in accordance with the principles of the Basel Declaration and the recommendations of the guidelines of The Animal Care & Welfare Committee, Institute of Materia Medica, Chinese Academy of Medical Sciences & Peking Union Medical College. The protocol was approved by The Animal Care & Welfare Committee, Institute of Materia Medica, Chinese Academy of Medical Sciences & Peking Union Medical College.

### Agents

IMMH001, IMMH001-P, FTY720, and FTY720-P were synthesized as previously described (Tian et al., 2013). The purities of IMMH001 and FTY720 were >98%, and those of IMMH001-P and FTY720-P were >95%. S1P was obtained from Sigma-Aldrich (St Louis, MO, USA) and the purity was >95%. MTX was purchased from Shanghai XinYi Pharmaceutical Co., Ltd. (Shanghai, China). All the compounds were dissolved in DMSO for *in vitro* assays.

### β-Arrestin Assay

The β-arrestin assay was performed by DiscoverX (Fremont, CA, USA) using enzyme fragment complementation with β-galactosidase as the functional reporter. PathHunter CHO-K1-EDG1, EDG3, EDG5, EDG6, and EDG8 cells were cultured in a total volume of 20 μL in white-walled, 384-well microplates and incubated at 37°C overnight. 5 μL of 5× samples with different concentration were added to the cells and incubated at 37°C for 2–4 h. Assay signals were generated by a single addition of 12.5 μL of PathHunter Detection reagent cocktail. The microplates were read following signal generation with a microplate reader (EnVision, PerkinElmer, Waltham, MA, USA) for chemiluminescent signal detection and the compound activities were analyzed using the CBIS data analysis suite (ChemInnovation Software, Inc., San Diego, CA, USA).

### Flow Cytometry Analysis of Lymphocyte Distribution

F344 rats were orally administered IMMH001 at a dose of 3 mg/kg. After 12 h, 100 μL of orbital plexus vein blood was collected from each rat and placed in heparinized centrifuge tubes. The red blood cells were removed according to the red blood cell lysate instructions and the remaining cells were resuspended with PBS containing 1% bovine serum albumin (BSA). The submandibular lymph nodes (SLN), axillary lymph nodes (ALN), mesenteric lymph nodes (MLN), Peyer’s patches (PP), spleens, and thymuses were dissected from the rats and homogenized into single cells. Tissue samples (1 × 10^6^ cells) were obtained and resuspended with PBS containing 1% BSA. All samples were blocked with 3% BSA and incubated with anti-CD45-647 (0.25 μg), anti-CD3-FITC (0.25 μg), and anti-CD45RC-PE (0.06 μg) antibodies for 1 h followed by fluorescence-activated cell sorting.

### AA Model

Lyophilized adjuvant bacillus Calmette-Guerin (BCG) vaccine was dissolved in distilled water at a concentration of 20 mg/kg. The solution was emulsified in an equal volume of complete Freund’s adjuvant (CFA) by ultrasonication in an ice water bath. AA was induced by intradermal injection in the right hind foot plantar with a total of 0.1 mL of the cold emulsion containing 1 mg BCG.

### CIA Model

Bovine type II collagen was dissolved in 0.1 M acetic acid at a concentration of 4 mg/mL at 4°C overnight. The solution was emulsified in an equal volume of CFA by ultrasonication on dry ice. On day 0, the rats were immunized intradermally in the right hind foot plantar with a total of 0.1 mL of the cold emulsion containing 2 mg of bovine type II collagen. On day 7, the rats subcutaneously received a booster injection at the base of the tail with 0.1 mL of the emulsion. Normal saline was injected into the rats as vehicle control following the procedure above.

### Administration of Drugs and Evaluation of the Anti-Arthritis Effect of the Drugs

All the drugs were dissolved in sterile water and were orally administered from the day of the CFA injection or day 9 after the first collagen injection. FTY720 (1 mg/kg) and IMMH001 (0.3, 0.6, 1.2, or 2.4 mg/kg) were given daily and MTX (0.5 mg/kg) was given twice a week for 28 days (AA) and 30 days (CIA), separately. For the vehicle, AA, and CIA control groups, the rats were given an equal volume of the solvent. All the rats were monitored daily for the onset of arthritis characterized by edema and erythema of the paws. The occurrence of arthritis and the effects of the drugs were assessed every 3–7 days following induction of arthritis by measuring body weight and paw size (perimeter and volume), as well as using an arthritic scoring system (arthritis index), which methods were widely used in similar studies (Matsuura et al., 2000; Pal et al., 2015; Yang et al., 2016). Paw swelling was calculated as the mean increase in the size and thickness of both hind paws measured with a plethysmometer (YLS-7A, Shandong Medical School, China) and a digital Vernier caliper, respectively. The arthritis index was scored on the following scale (Larsson et al., 1990): 0 = no signs of arthritis; 1 = slight edema and erythema in the foot or ankle; 2 = slight edema and erythema in the entire paw; 3 = moderate edema and erythema in the entire paw; 4 = severe edema and ankyloses, inability to walk. Each paw was graded, and the severity score was the sum of the scores of each paw with the maximum score being 16.

### Peripheral Blood Lymphocyte Counts

On day 28 of the AA experiments and day 38 of the CIA experiments, 20 μL blood samples were collected from the tail vein and measured with a blood cell analyzer (MEK-7222K, Nihon Kohden, Tokyo, Japan).

### X-Ray Analysis

The bones of the rats were fixed with 4% polyformaldehyde and scanned with a CT system (INVEON MM, Siemens, Munich, Germany) for 20 ms. The voltage of the X-ray bulb tubes was set to 80 kV and the current was 500 mA. The control group was used as a reference for the degree of injury. The following parameters were evaluated on a scale of 1–3 to determine the injury severity quantitatively: soft tissue swelling in the paws; osteoporosis; bone erosion; gap reduction; and joint injury and damage to the arrangement structure. The degree of osteoporosis was based on the X-ray penetration rate; bone erosion was based on the degree of bone tissue destruction, irregular new osteophytes in the cortical bone, surface roughness, and damage to the normal cortical bone; the gap reduction and joint injury and damage to the arrangement structure were based on whether the joint spaces were narrowed or fused in the rat paw images. It was also considered whether the positions of the bones and joints were anatomically normal. The full score was 15 points.

### Cytokine Detection

Cytokines were analyzed with a multi-factor kit (Millipore, Burlington, MA, USA) according to the manufacturer’s instructions. The blank plate was first washed with 1× wash buffer (200 μL) and vortexed for 10 min at room temperature. Assay buffer (25 μL) and the sample (25 μL; serum and 1 μg/µL articular tissue protein lysate) were added to the sample wells. Beads (25 μL) were added and the mixture was vortexed before it was placed in the wells. The plate was sealed and put on a plate shaker at 500 rpm overnight at 2–8°C in the dark. The plate was incubated at room temperature for 30 min, put on a magnet for 60 s, and then the supernatant was gently removed before washing. The plate was washed twice with wash buffer (200 μL) and shaken at 350 rpm for 2 min. Detection antibodies (25 μL) were added to each well and the plate was incubated on a plate shaker at 650 rpm for 1 h at room temperature in the dark. Streptavidin–phycoerythrin (25 μL) was added to the wells and the plate was incubated under the same conditions for 30 min and washed twice. Sheath fluid (150 μL) was added to all wells and the plate was shaken at 350 rpm for 5 min. Finally, the plate was read with a plate reader (Luminex 200, Luminex, Austin, TX, USA) with xPONENT software (Luminex). Results were analyzed by MILLIPLEX Analyst 5.1 (Millipore).

### Histology and Hematoxylin and Eosin Staining

The primary side of the rat hind paws was removed at postmortem and fixed in 4% paraformaldehyde for hematoxylin and eosin staining at the end of the study. The histological score for arthropathy, also called the pathological score, was calculated as the sum of inflammatory cell infiltration, synovial hyperplasia, pannus formation, and bone destruction. The score was evaluated by a professional histologist in a double blind fashion as follows. Inflammatory cell infiltration: 0 = no inflammatory cells; 1 = some inflammatory cells; 2 = moderate number of inflammatory cells; 3 = abundant inflammatory cells. Synovial hyperplasia: synovial epithelial cell proliferation = 1; fibrous tissue hyperplasia = 1; inflammatory cell infiltration = 1. Pannus formation: 0 = no pannus; 1 = slight pannus; 2 = moderate pannus; 3 = large pannus. Bone destruction: 0 = no destruction; 1 = slight destruction, cortical bone destruction, cortical bone thickness is less than half normal thickness; 2 = moderate destruction, apparent resorption of trabecular and cortical bone, but not full bone cortex; 3 = severe destruction, destruction to full bone cortex, cortical deformation, and trabecula bone absorption.

### Statistical Analysis

All data is presented as mean ± standard error. Analyses were performed using the *t*-test (for unpaired two-group analysis) and the one-way ANOVA test (for three or more groups).

## Results

### IMMH001-P Activated S1P_1_ *In Vitro*

Similar to FTY720, IMMH001 is a prodrug that is phosphorylated to form IMMH001-P, which stimulates S1P_1_ but not S1P_3_ *in vitro*. Therefore, we tested the selectivity of IMMH001-P on different S1P_1-5_ subtypes using a β-arrestin assay. S1P_1_, S1P_4_, and S1P_5_ were activated by IMMH001-P with EC_50_ values of 12.4, 19.8, and 29.4 nM, respectively (**Table 1**). However, the effects of IMMH001-P on S1P_2_ and S1P_3_ were much weaker, with EC_50_ values of more than 1,000 nM. Thus, IMMH001-P is a good modulator for S1P_1_, S1P_4_, and S1P_5_.

**Table 1:** The activation effects of IMMH001 and IMMH001-P on S1P_1-5_.

Receptor	EC_50_ (nM)		
	IMMH001	IMMH001-P	S1P
S1P_1_	749.9	6.1	31.8
S1P_2_	>10000	2862.4	76.1
S1P_3_	2276.3	>5000	15.2
S1P_4_	211.9	16.7	149.3
S1P_5_	466.7	14.8	31.2

### IMMH001 Regulates Lymphocyte Distributions

**Figure 1:**
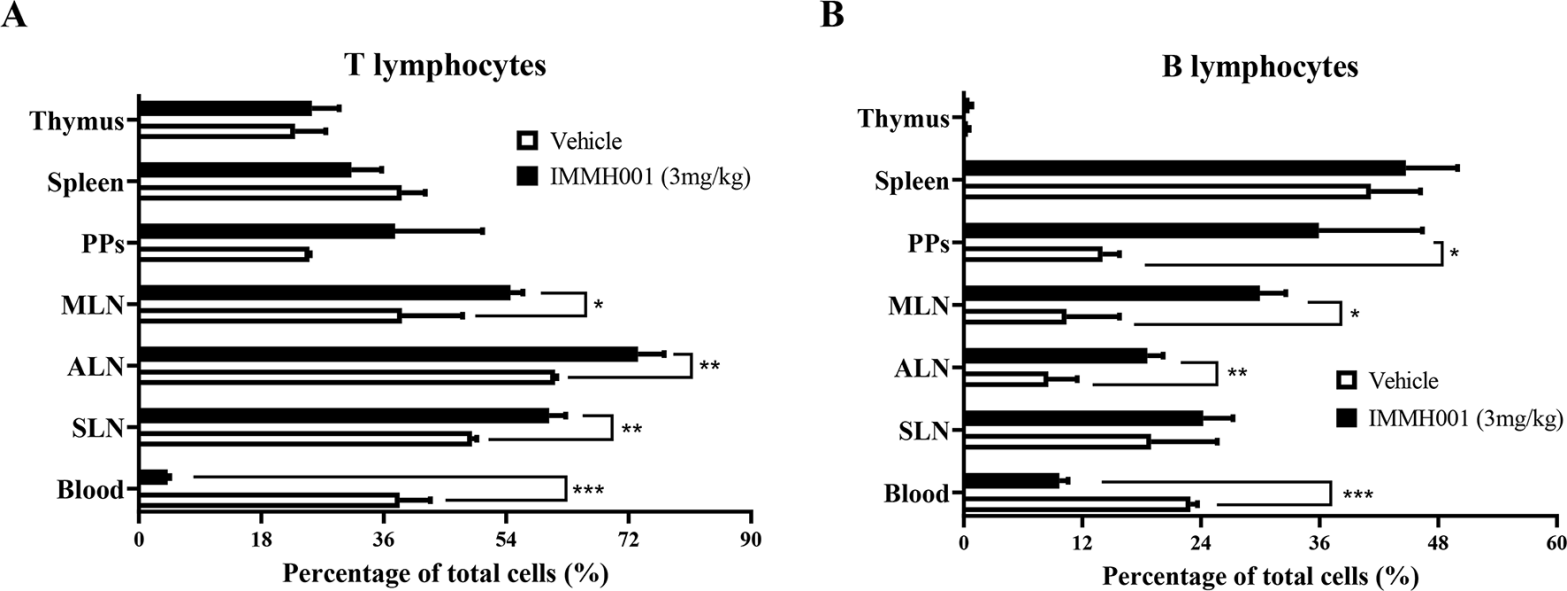
Lymphocyte redistributions by IMMH001. The percentage of T lymphocytes **(A)** and B lymphocytes **(B)** in different lymph tissue and blood after 12 h administration were determined by flow cytometry. Each symbol represents the mean ± SEM of six F344 rats. *P < 0.05, **P < 0.01, ***P < 0.001.

As a novel S1P_1_ modulator, we previously demonstrated that IMMH001 decreased the number of peripheral blood lymphocytes in Sprague-Dawley rats following a single oral dose. In the present study, we determined the distribution of these peripheral blood lymphocytes in greater detail. In F344 rats, we observed that the peripheral blood lymphocytes (T lymphocytes and B lymphocytes) were significantly decreased by a single dose of IMMH001, and that the lymphocytes in secondary lymphoid tissues (SLN, ALN, MLN, and PP) were increased (**Figure 1** and **Supplementary Figure 1**). The trend of total white blood cells of rats after administration of IMMH001 was similar to lymphocytes (**Supplementary Figure 2**). These results demonstrated that IMMH001 affected lymphocyte distribution and induced peripheral blood lymphocyte homing to secondary lymphoid tissues.

### IMMH001 Exhibits a Therapeutic Effect in the AA Rat Model

**Figure 2:**
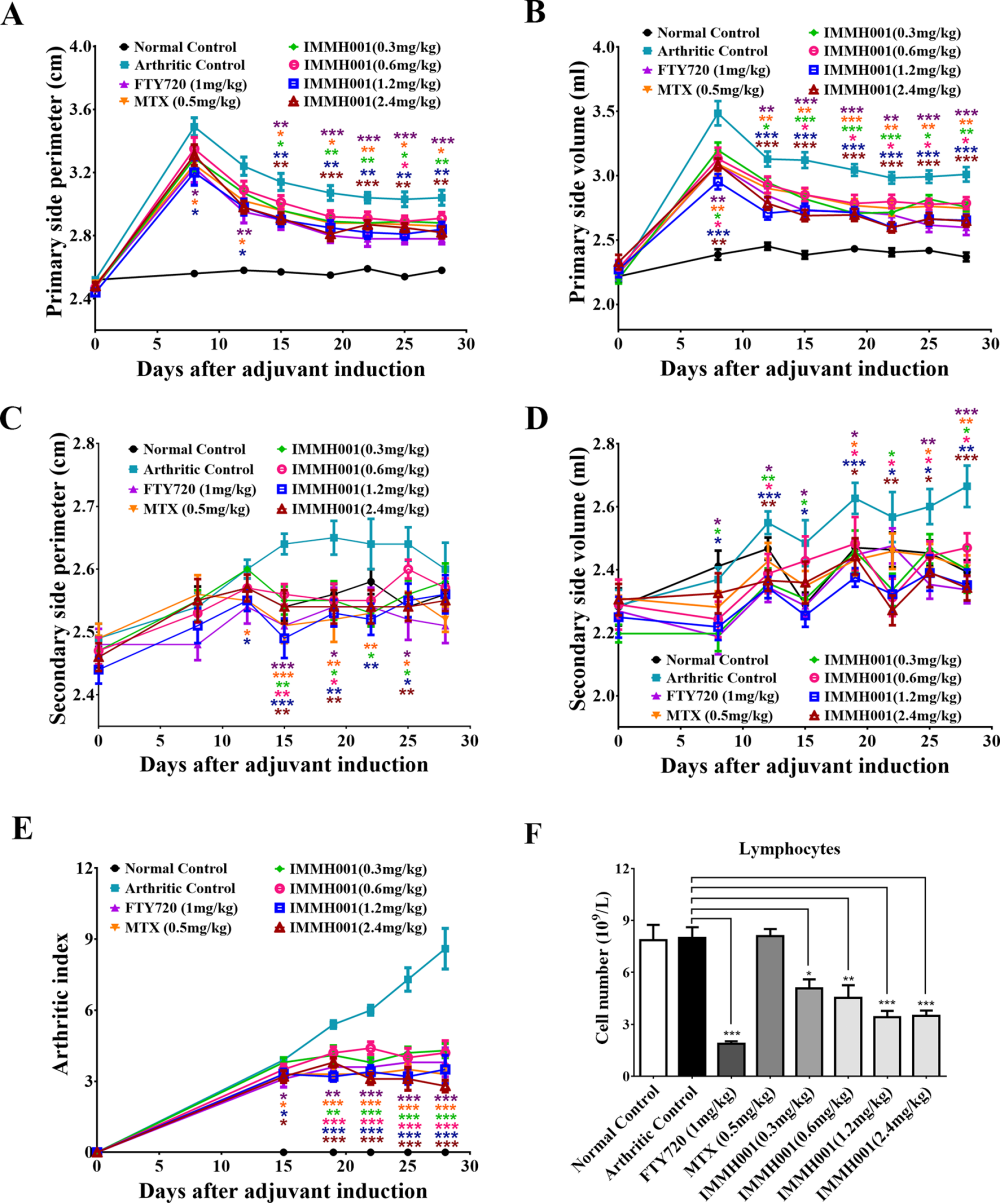
IMMH001 decreased hind paw perimeter, volumes and arthritis index of AA rats. At the 0, 8, 12, 15, 19, 22, 25, 28 day of IMMH001 administration, the primary hind paw perimeter **(A)** and volume **(B)** were measured. Meanwhile, the secondary hind paw perimeter **(C)** and volume **(D)** were also determined. At the 0, 15, 19, 22, 25 and 28 day, the arthritis indexes of different groups of AA rats were evaluated according to the criteria **(E)**. **(F)** IMMH001 reduced circulating lymphocytes on AA rats. After 28 days of IMMH001 treatment, the lymphocytes from the tail vain blood were determined by MEK-7222K type blood cell analyzer. Each symbol represents the mean ± SEM of ten rats. *P < 0.05, **P < 0.01, ***P < 0.001 versus arthritic control. Each symbol represents the mean±SEM of ten animals.

**Figure 3:**
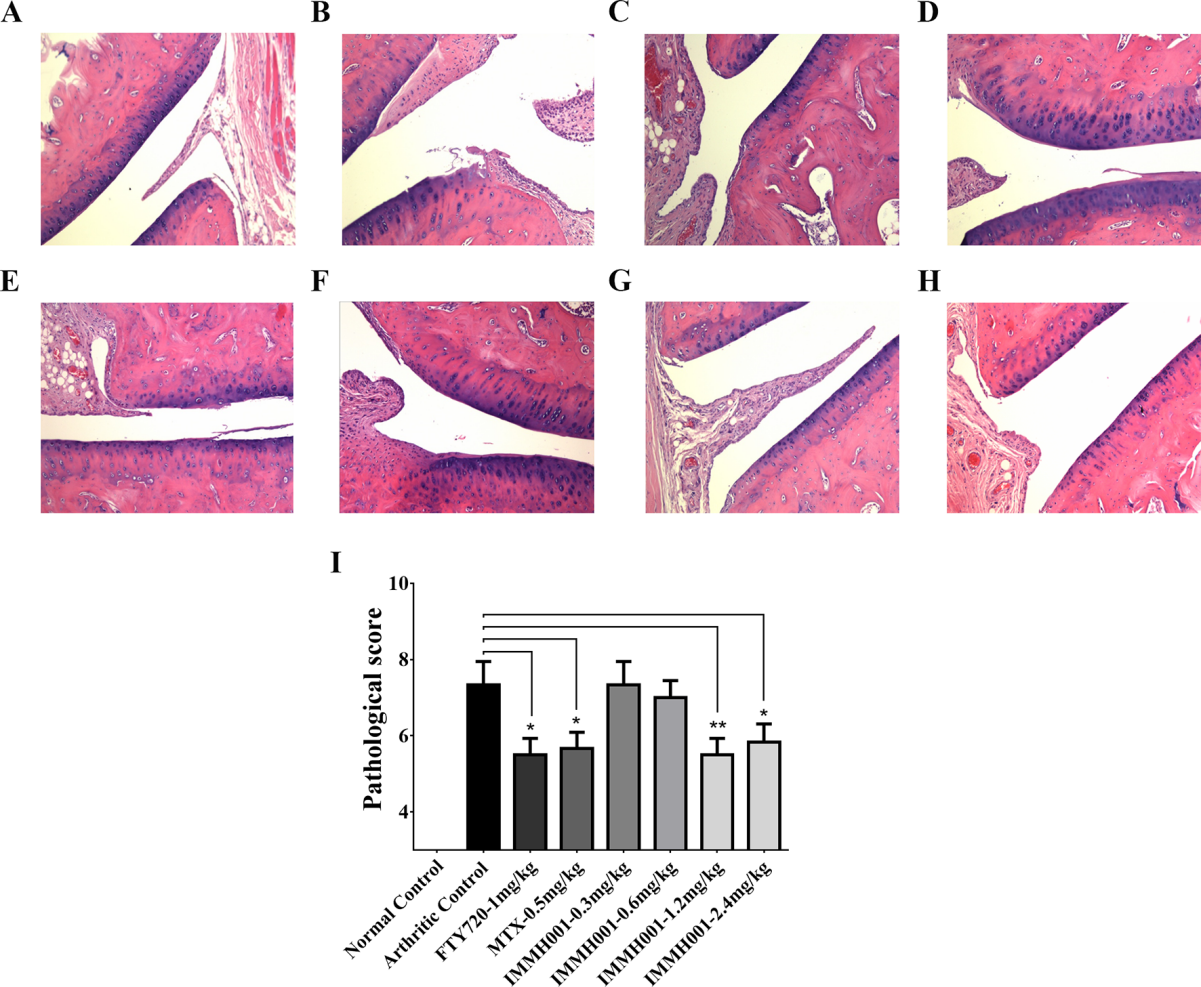
IMMH001 relieved the damage of AA rats’ joints. The primary side of AA rats’ joints were obtained on day 28 after adjuvant injection, and HE staining **(A**–**H)** and histological score **(I)** were performed. The statistical graph of histological score represented the damage in rat joints. Data were expressed as mean ± SEM of six animals. *P < 0.05, **P < 0.01 versus arthritic control. A representative HE staining photo of articular cavity at six different rats was shown: **(A)** Normal control rats treated with vehicle, **(B)** AA control rats treated with vehicle, **(C)** AA rats treated with 1mg/kg FTY720, **(D)** AA rats treated with 0.5 mg/kg MTX, **(E)** AA rats treated with 0.3 mg/kg IMMH001, **(F)** AA rats treated with 0.6 mg/kg IMMH001, **(G)** AA rats treated with 1.2 mg/kg IMMH001, **(H)** AA rats treated with 1.2 mg/kg IMMH001. The magnification was ×100.

RA is characterized by immune cell infiltration with proinflammatory cytokine release. Because IMMH001 induced peripheral lymphocyte homing to lymphoid tissues, we questioned whether IMMH001 could reduce the infiltrated lymphocytes in the RA region and be beneficial for RA therapy. Therefore, we explored the effect of IMMH001 on AA. As expected, the hind paw perimeter and primary side volume of the arthritic control rats increased from the day of the adjuvant injection (**Figures 2A, B**). Subsequently, the hind paw perimeter and secondary side volume increased on day 8 after the adjuvant injection (**Figures 2C, D**). The positive controls, FTY720 and MTX, inhibited the swelling of the hind paws on both sides at doses of 1 mg/kg. These two drugs also showed a strong inhibitory effect on the arthritis index (**Figure 2E**). Interestingly, IMMH001 also showed a dose-dependent inhibition effect on the primary and secondary side perimeter and volume and the total arthritis index. This effect at doses of 1.2 or 2.4 mg/kg was comparable to those of FTY720 and MTX. Long-term administration of IMMH001 for 28 consecutive days reduced blood lymphocytes (**Figure 2F**). At the end of the experiments (day 28), all animals were sacrificed for histopathological examinations. Representative histological images from the primary side ankle joints of the rats are shown in **Figures 3A–H**. In the joint tissue of normal, healthy rats, the cartilage and synovial cavity were intact, and there were few nucleated cells (**Figure 3A**). In contrast, inflammatory cell infiltration, synovial hyperplasia, and severe pannus formation were observed in the synovial joints of the untreated AA rats (**Figure 3B**). The AA rats treated with MTX, FTY720, and IMMH001 all showed much less damaged ankle joints compared with the untreated rats (**Figures 3C–H**). The total pathological score analysis indicated that the score was 7.3 ± 1.5 in the sections of joint tissue of the untreated AA rats, whereas IMMH001 significantly reduced the score to 5.5 ± 1.0 and 5.8 ± 1.2 at doses of 1.2 and 2.4 mg/kg, respectively, which was a similar reduction to MTX (5.7 ± 1.0) and FTY720 (5.5 ± 1.0) (**Figure 3I** and **Supplementary Table 1**).

**Figure 4:**
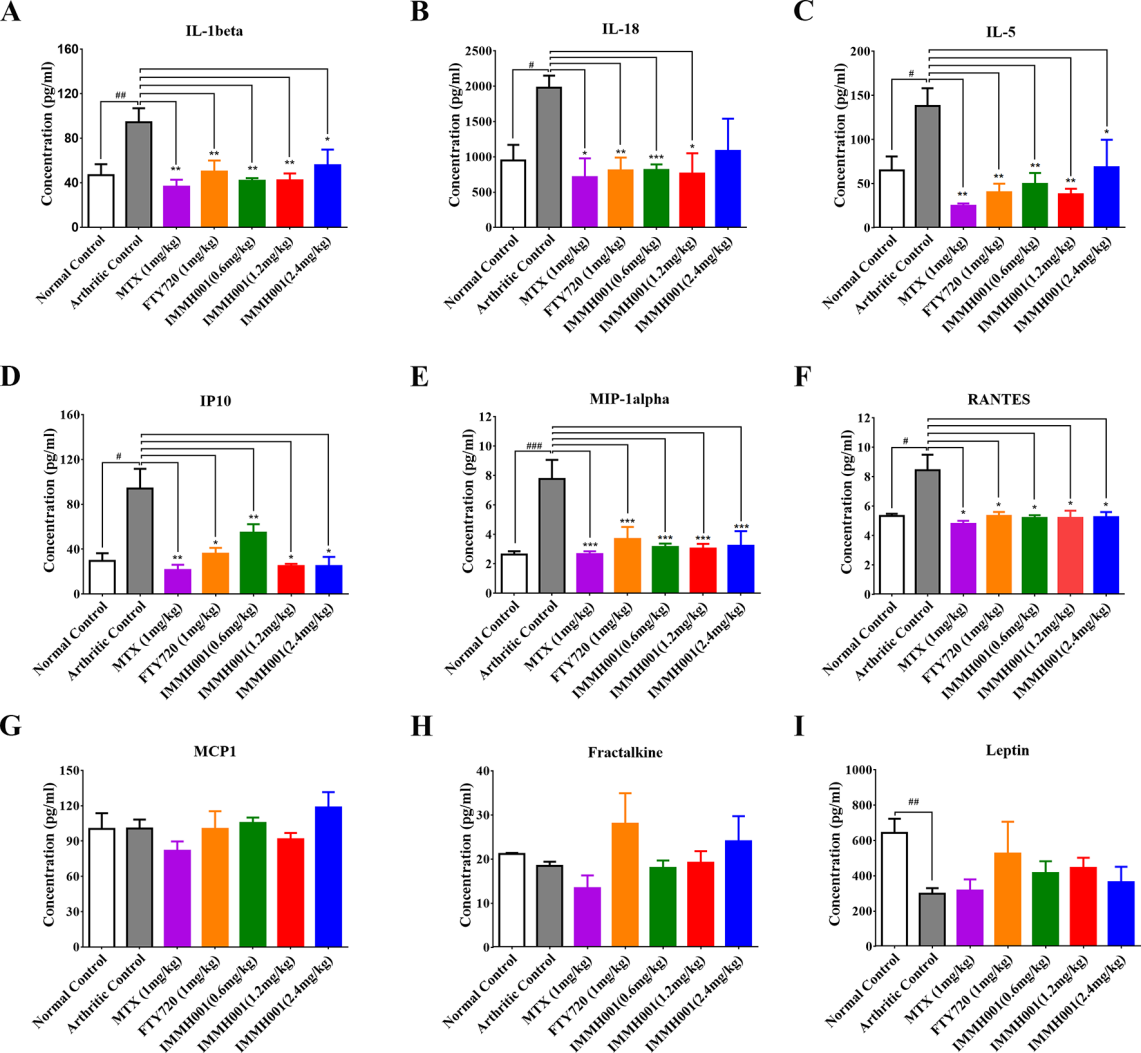
IMMH001 reduced the levels of proinflammatory cytokines and chemokines of AA rats’ joints. The rats’ joints was collected in the end of experiment. **(A)** interleukin-1 beta (IL-1 beta), **(B)** interleukin-18 (IL-18), **(C)** interleukin-5 (IL-5), **(D)** Interferon-gamma-induced protein 10 (IP10), **(E)** Macrophage inflammatory protein-1alpha (MIP1-alpha), **(F)** regulated on activation, normal, T-cell expressed, and secreted (RANTES), **(G)** monocyte chemotactic protein 1 (MCP-1), **(H)** Fractalkine, and **(I)** Leptin. Data were indicated as mean ± SEM of four animals.

We also collected joint tissues and serum for cytokine detection. Proinflammatory cytokines and chemokines, such as interleukin (IL)-1β, IL-5, IL-18, chemokine ligand (CCL) 3 (also called macrophage inflammatory protein 1-α), CCL5 (also known as RANTES), and interferon gamma-inducible protein 10 (IP10), in joints were elevated dramatically in the AA model compared with the normal control rats, whereas IMMH001 significantly decreased these proinflammatory cytokines and chemokines in the AA model (**Figures 4A–F**). Other cytokines, such as CCL2 [also known as monocyte chemoattractant protein (MCP)-1], fractalkine, and leptin, were not affected by IMMH001 treatment (**Figures 4G–I**). Importantly, these cytokines and chemokines were not changed in the serum after IMMH001 treatment (**Supplementary Figure 3**).

### IMMH001 Ameliorates CIA

**Figure 5:**
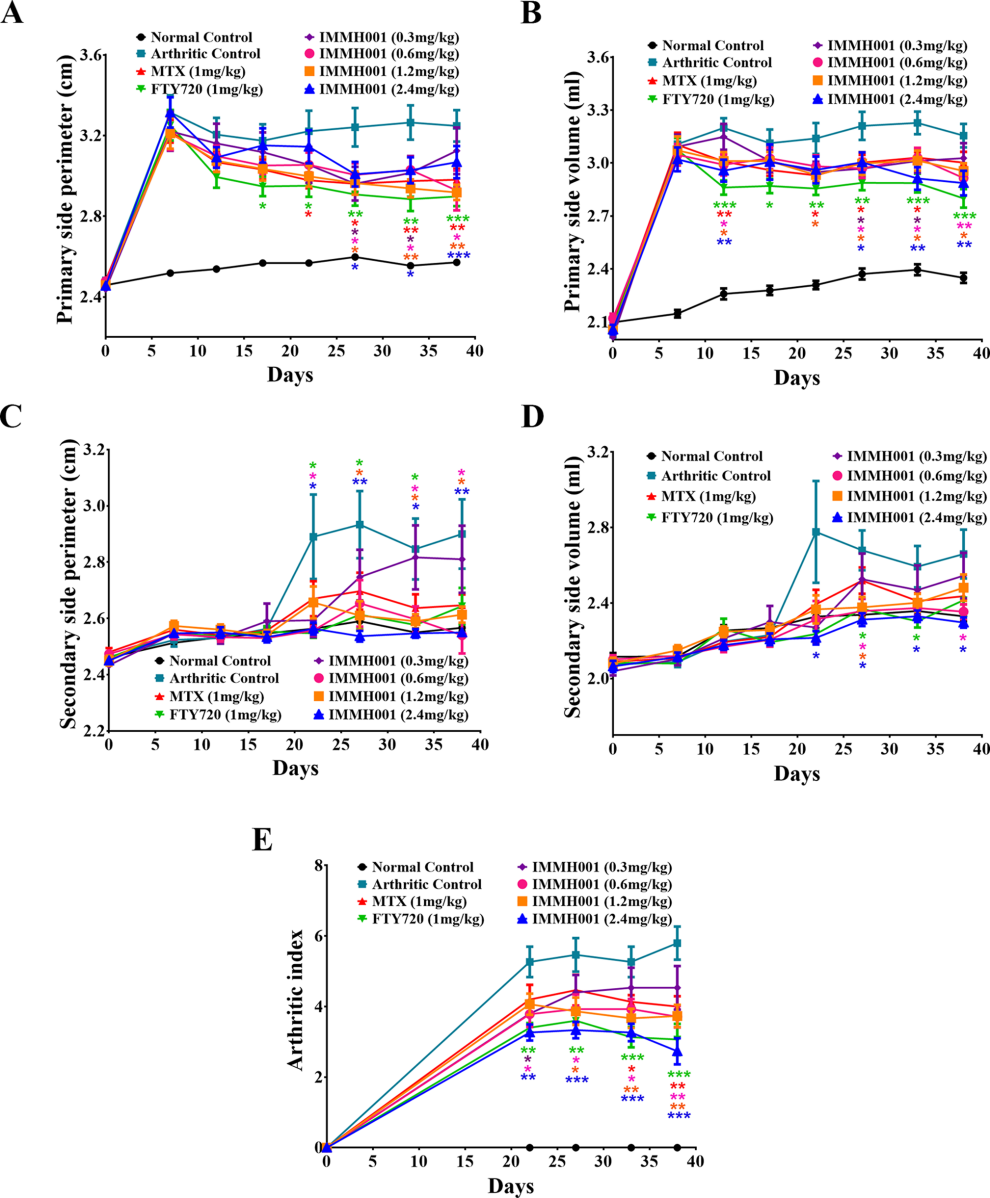
IMMH001 decreased hind paw perimeter, volumes and arthritis index of CIA rats. At the 0, 7, 12, 17, 22, 27, 33, 38 day of IMMH001 administration, the primary hind paw perimeter **(A)** and volume **(B)** were measured. Meanwhile, the secondary hind paw perimeter **(C)** and volume **(D)** were also determined. At the 0, 22, 27, 33 and 38 day, the arthritis indexes of different groups of CIA rats were evaluated according to the criteria **(E)**. Each symbol represents the mean ± SEM of ten animals.

**Figure 6:**
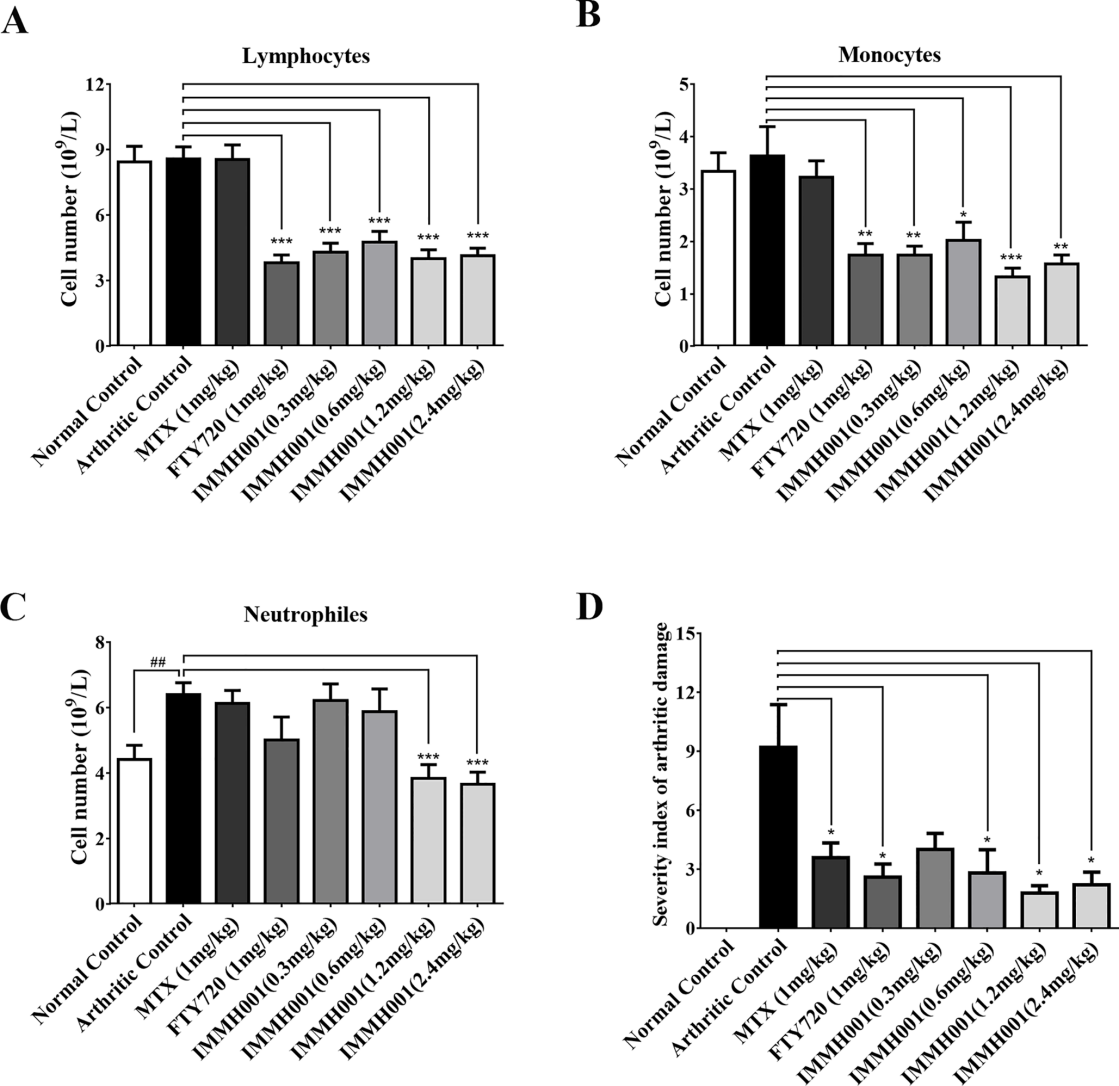
IMMH001 reduced circulating lymphocytes and joints damage on CIA rats. After 38 days of IMMH001 treatment, the lymphocytes **(A)**, monocytes **(B)** and neutrophils **(C)** from the tail vain blood were determined by MEK-7222K type blood cell analyzer. Each symbol represents the mean ± SD of ten rats. **(D)** Severity index of arthritic damage based on X-ray analysis. Each symbol represents the mean ± SD of five rats.*P < 0.05, **P < 0.01, ***P < 0.001 versus arthritic control.

**Figure 7:**
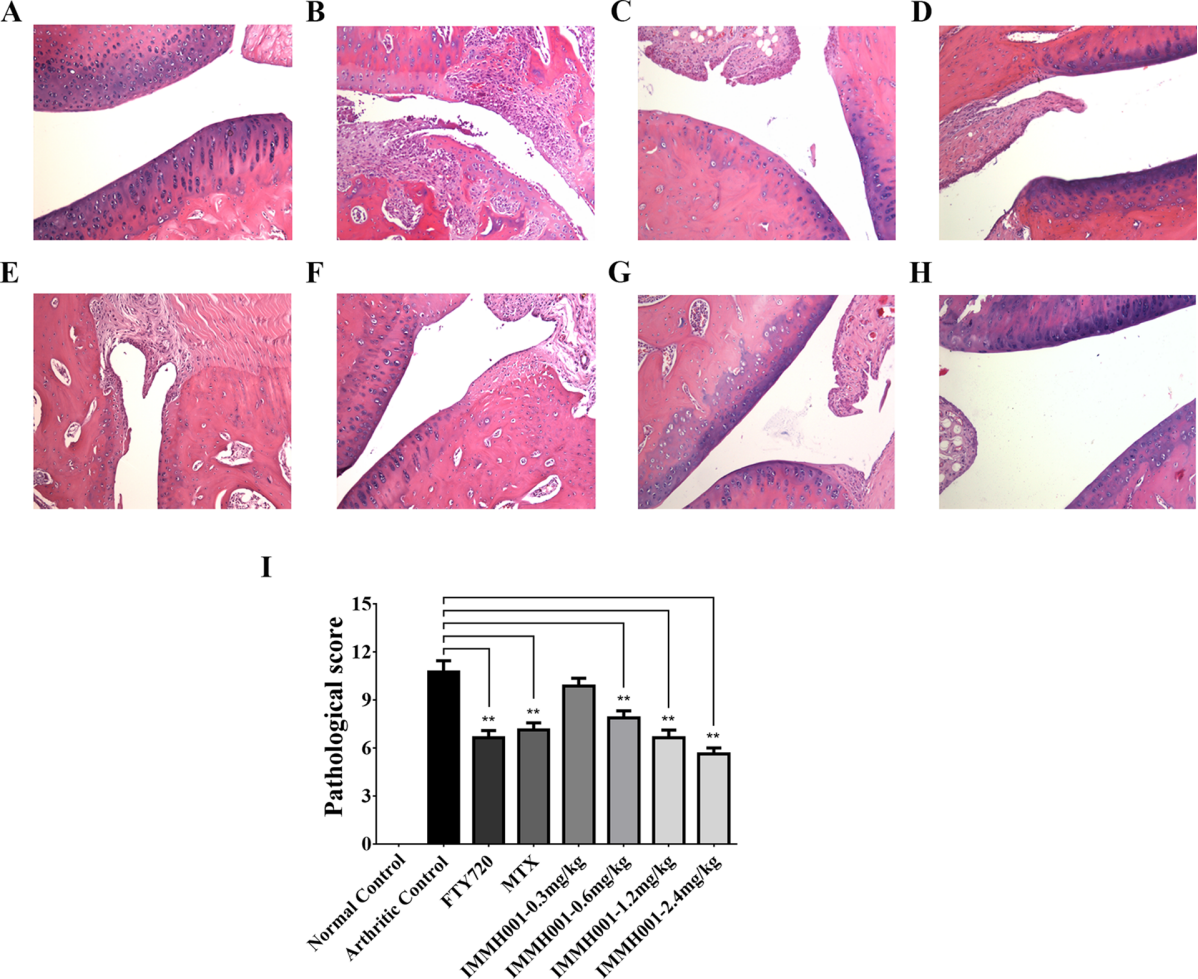
IMMH001 relieved the damage of CIA rats’ joints. The primary side of CIA rats’ joints were obtained on day 38 after collagen injection, and HE staining **(A**–**H)** and histological scoring **(I)** were performed. The statistical graph of histological score represented the damage in rat joints. Data were expressed as mean ± SD of six animals. *P < 0.05, **P < 0.01 versus arthritic control. A representative HE staining photo of articular cavity at six different rats was shown: **(A)** Normal control rats treated with vehicle, **(B)** CIAcontrol rats treated with vehicle, **(C)** CIA rats treated with 1mg/kg FTY720, **(D)** CIA rats treated with 0.5 mg/kg MTX, **(E)** CIA rats treated with 0.3 mg/kg IMMH001, **(F)** CIA rats treated with 0.6 mg/kg IMMH001, **(G)** CIA rats treated with 1.2 mg/kg IMMH001, **(H)** CIA rats treated with 1.2 mg/kg IMMH001. The magnification was ×100. Arrow a pointed to inflammatory cell infiltration, arrow b pointed to synovial hyperplasia, arrow c pointed to pannus formation and arrow d pointed to bone destruction.

The CIA rat model is another well-defined arthritis animal model. Seven days after the collagen injection, prominent articular swelling (hind paw perimeter and volumes) of the arthritic control rats’ primary hind paws was observed (**Figures 5A, B**), whereas articular swelling of the secondary hind paws was observed on day 17 after injection (**Figures 5C, D**). Surprisingly, IMMH001 ameliorated the articular swelling at all doses from 0.3 to 2.4 mg/kg, which was comparable to MTX and FTY720 (**Figure 5**). Furthermore, IMMH001 decreased the total arthritis index, similar to FTY720 and MTX. After 38 consecutive days of IMMH001 oral administration, blood levels of lymphocytes, monocytes, and neutrophils were reduced (**Figures 6A–C**). X-ray examination of the joints showed that the severity index of the arthritic damage was significantly rescued by IMMH001 (**Figure 6D** and **Supplementary Figure 4**). Histopathological examination of the rat joints showed inflammatory cell infiltration, synovial hyperplasia, and severe pannus formation in the synovial joints of the untreated CIA rats, but not in the normal control tissues (**Figures 7A, B**). IMMH001 treatment significantly ameliorated this joint damage, similar to MTX and FTY720 (**Figures 7C–H**), as confirmed by the markedly reduced inflammatory cell infiltration in the IMMH001 treatment groups. The total pathological score for the sections of CIA control joint tissue was 10.8 ± 2.0, whereas IMMH001 doses of 0.6, 1.2, and 2.4 mg/kg significantly reduced the scores to 7.9 ± 1.2, 6.6 ± 1.4, and 5.6 ± 1.1, respectively, which were similar to the reductions produced by MTX (7.1 ± 1.2) and FTY720 (6.6 ± 1.3) (**Figure 7I** and **Supplementary Table 2**).

## Discussion

RA is a chronic, systemic autoimmune disease characterized by inflammation and destruction of the joints and progressive disability. Infiltrated cells including granulocytes, monocytes/macrophages, natural killer (NK) cells, B cells, and especially T cells have been implicated in the pathogenesis of RA by producing many chemokines and proinflammatory cytokines (Drexler et al., 2008; Mellado et al., 2015, Wehr et al., 2018; Yap et al., 2018). Although the roles of these inflammatory cells is poorly understood, blocking or inhibiting these cells that secrete proinflammatory cytokines relieves the symptoms of RA (Bullock et al., 2018; Mahajan and Mikuls, 2018). For example, both the Janus kinase 3 inhibitor tofacitinib, which decreases the number of CD16/56^+^ NK cells, and the tumor necrosis factor (TNF)-α inhibitor etanercept are widely used for clinical RA therapy (Zhao et al., 2018).

The sphingolipid S1P_1_ regulat es the trafficking of a variety of immune cells. Activation of S1P_1_ recirculates lymphocytes from peripheral to secondary lymphoid organs, reducing the peripheral immune response. Previously, we have shown that IMMH001 (Syl930) had a great agonist activity on S1P1 and could decrease lymphocytes in periphery blood in a dose dependent manner (Jin et al., 2014). In the present study, we found that besides reducing peripheral blood lymphocytes of animals, IMMH001 could increase the lymphocytes in secondary lymphoid organs at the same time, which further confirmed the modulation effect of IMMH001 on lymphocytes trafficking. In addition, we have shown that oral administration of IMMH001 exerted potential therapeutic effects in experimental allergic encephalomyelitis (Jin et al., 2014) and psoriasis animal models (Ji et al., 2018). Moreover, it was more specific than the launched S1P_1_ agonist FTY720 on S1P_3_, which receptor maybe correlated with the risk of bradycardia in patients (Forrest et al., 2004; Sanna et al., 2004) and has better pharmacokinetic properties, such as a shorter half-life, which could make the lymphocytes recover soon after withdrawal once the patients had infection. Thus, it is conceivable that IMMH001 may be suitable for RA therapy. Here, we found that IMMH001 exhibited a significant therapeutic effect in both the AA and CIA models. IMMH001 alleviated the RA symptoms in the joints, decreased the inflammatory cell infiltration and synovial hyperplasia, reduced the release of proinflammatory cytokines, and finally improved the pathological status of damaged joints. These observations were consistent with the findings that FTY720 reduced CD4 positive T cells and improved pathological changes in the CIA model (Wang et al., 2007; Tsunemi et al., 2010).

The pathogenesis of RA is correlated with many cytokines, such as TNF-α, IL-1, IL-6, IL-17, and IL-18, and matrix metallopeptidases. The cytokine-mediated immune response plays a crucial role in the pathogenesis of RA and anti-cytokine therapy is beneficial for RA. TNF-α and IL-1β are key cytokines that induce cartilage damage during RA pathological progression. In addition, the synovial tissue and synovial fluid of RA patients contain increased concentrations of several chemokines, including CCL13 (also known as MCP-4), CCL2, CCL3, CCL5, and fractalkine. In our rat AA model, we found that in the damaged joints, IMMH001 decreased levels of both chemokines and proinflammatory cytokines, including IL-1β, IL-5, IL-18, IP10, CCL3, and CCL5, although CCL2, fractalkine, and lectin were not affected. These cytokines and chemokines were not significantly affected in the serum after IMMH001 treatment. The cytokine analysis suggested that IMMH001 suppressed both Th1 cell (IL-1β, IL-18, and IP10) and Th2 cell (IL-5)-mediated disease reactions in damaged joints. This might due to the effect of IMMH001 on whole lymphocytes, which was also observed in the FTY720 and MTX groups. MTX alters the cytokine profile in the damaged joints of RA patients (Friedman and Cronstein, 2018). However, we did not detect TNF-α or IL-1β in the serum of either RA model.

Our data demonstrates that IMMH001 showed significant therapeutic effects in RA animal models. IMMH001 is now in a phase I clinical trial for RA therapy in China.

## Data Availability

The raw data supporting the conclusions of this manuscript will be made available by the authors, without undue reservation, to any qualified researcher.

## Ethics Statement

All the rat experiments were in accordance with guidelines of the Committee on Animals of the Institute of Materia Medica, Chinese Academy of Medical Sciences & Peking Union Medical College.

## Author Contributions

JJ and MJ carried out most of the research work and wrote the manuscript. RF, MW, NX and JH helped in carrying out the animal research. QX and XW supported the compunds of this work. FL, DY and XC helped in designing the research and revising the paper.

## Funding

This work is supported by National Natural Science Foundation of China (NSFC Nos. 81872923 and 81473096), Beijing Natural Science Foundation (No. 7172140), and the CAMS Innovation Fund for Medical Sciences (2016-I2M-3-008).

## Conflict of Interest Statement

The authors declare that the research was conducted in the absence of any commercial or financial relationships that could be construed as a potential conflict of interest.
