# Supplementary material for: Sphingosine-1-Phosphate Receptor Subtype 1 (S1P1) Modulator IMMH001 Regulates Adjuvant- and Collagen-Induced Arthritis
Source: Front Pharmacol. 2019 Sep 19;10:1085. doi: 10.3389/fphar.2019.01085 (PMC6761374; doi:10.3389/fphar.2019.01085)
Supplement: Supplementary file 1 [file DataSheet_1.docx]

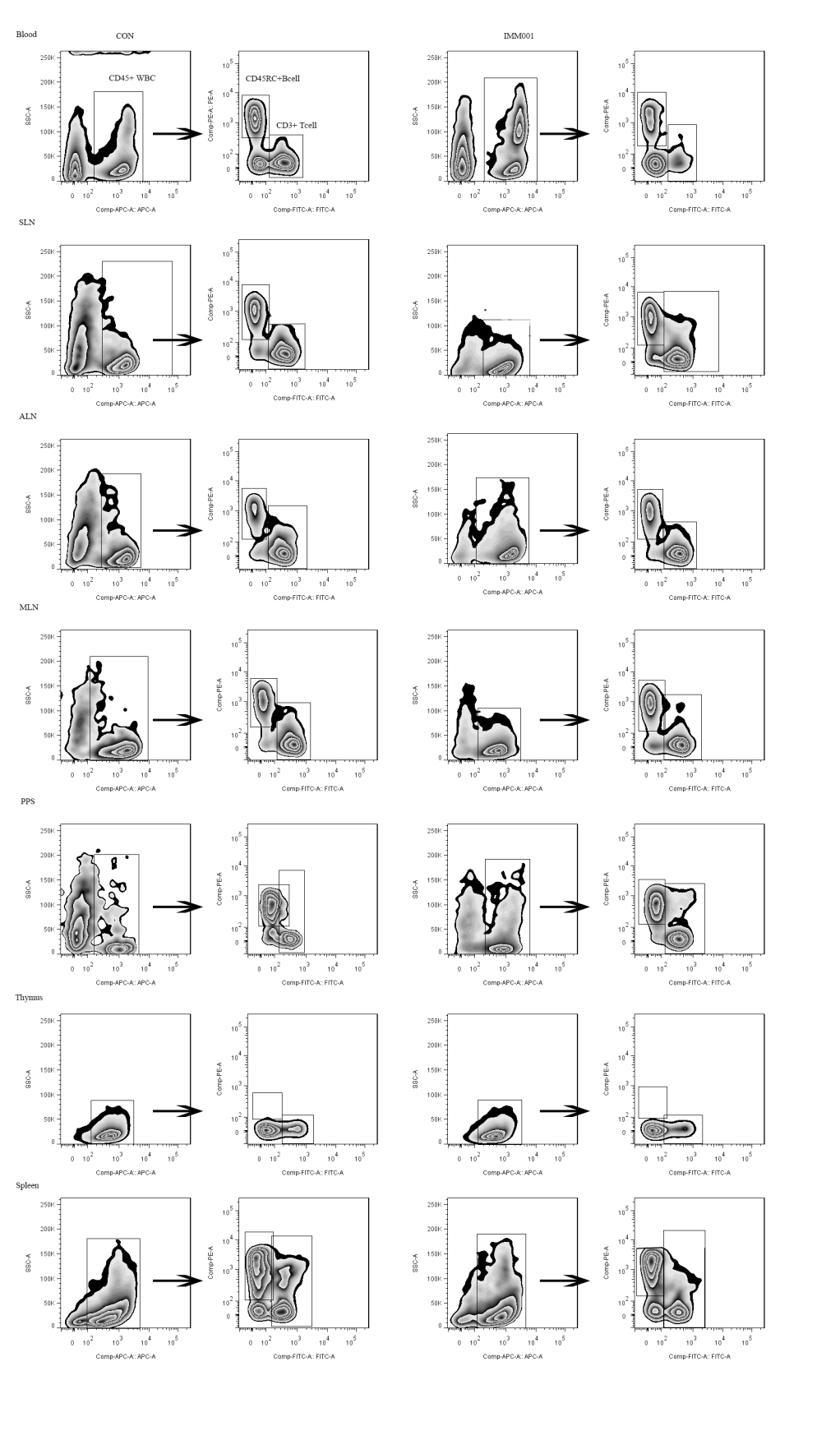


Supplementary Figure 1. Flow cytometry analysis of lymphocyte redistributions by IMMH001. The proportion of WBC was calculated by CD45+ cells in total live cells. The proportion of T lymphocytes was calculated by CD45+CD3+ cells in total live cells. The proportion of B lymphocytes was calculated by CD45+CD45RC+ cells in total live cells.


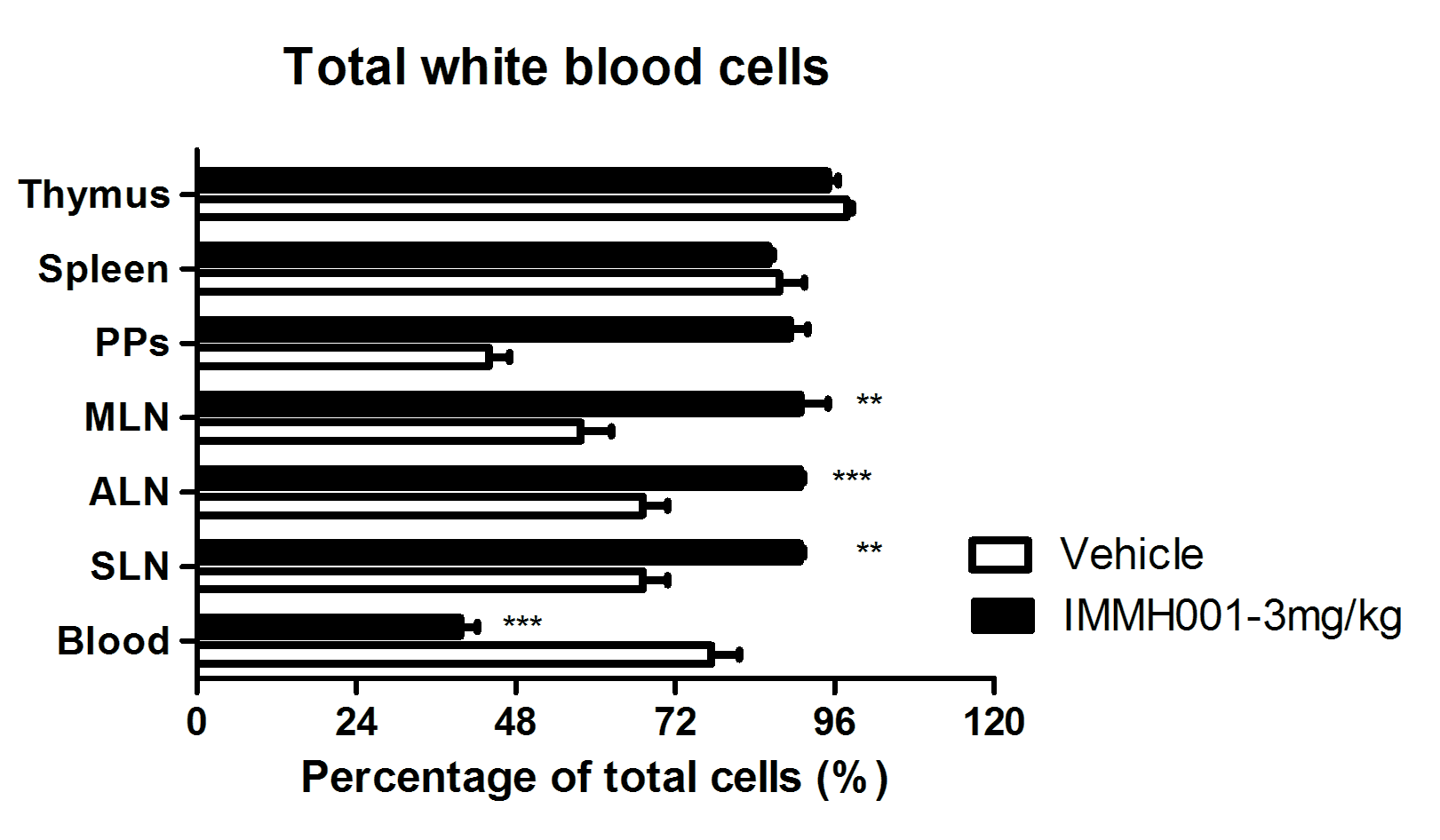


Supplementary Figure 2. WBC redistributions by IMMH001. The percentage of total white blood cells in different lymph tissue and blood after 12h administration were determined by flow cytometry. Each symbol represents the mean±SEM of six F344 rats. ^**^ P <0.01, ^***^ P <0.001.


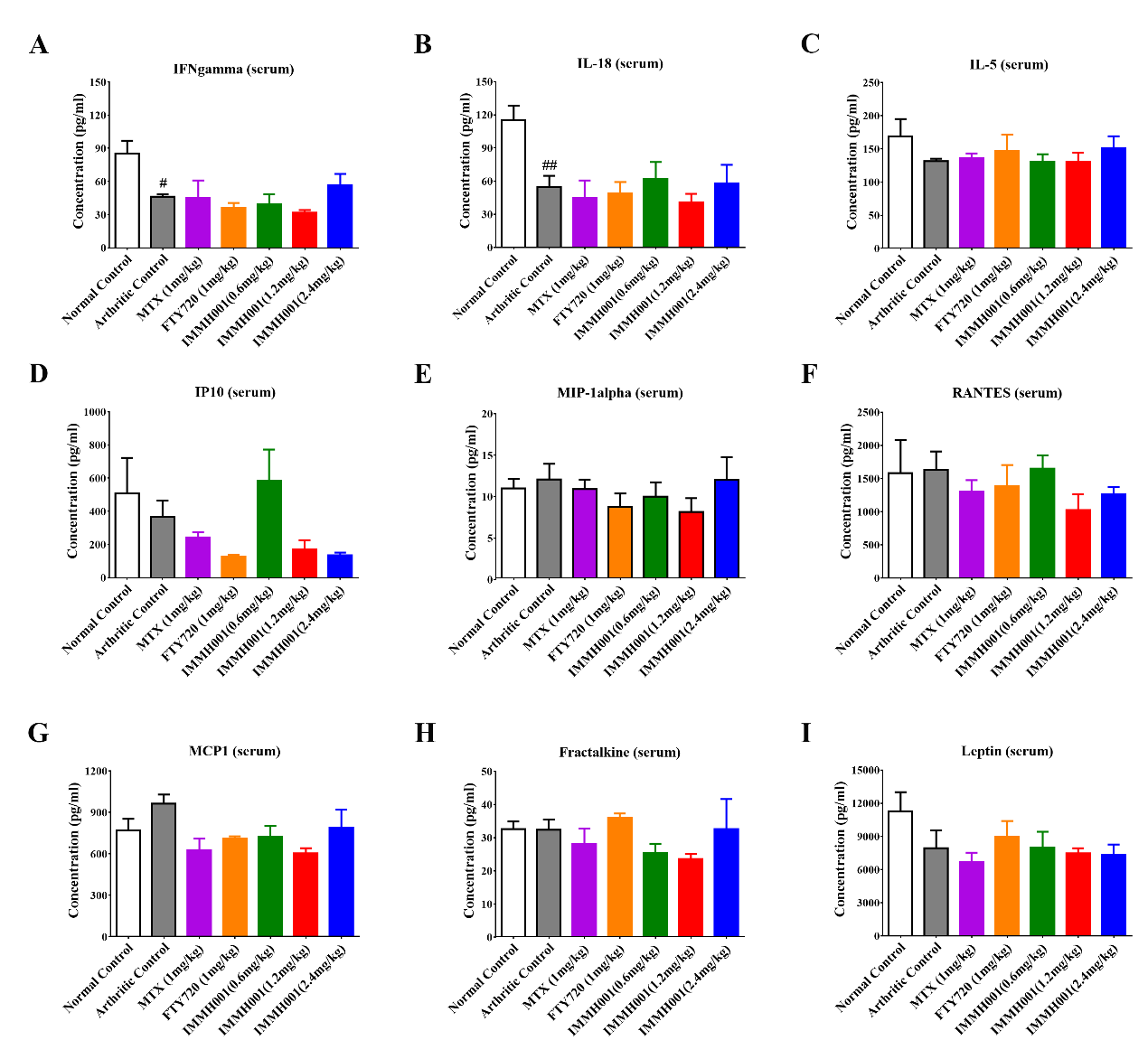


Supplementary Figure 3. The levels of proinflammatory cytokines and chemokines in AA rats’ serum. The rats’ serum was collected in the end of experiment. (A) Interferon-gamma (IFN-gamma), (B) interleukin-18 (IL-18), (C) interleukin-5 (IL-5), (D) Interferon-gamma-induced protein 10 (IP10), (E) Macrophage inflammatory protein-1alpha (MIP1-alpha), (F) regulated on activation, normal, T-cell expressed, and secreted (RANTES), (G) monocyte chemotactic protein 1 (MCP-1), (H) Fractalkine, and (I) Leptin. Data were indicated as mean±SEM of four animals.


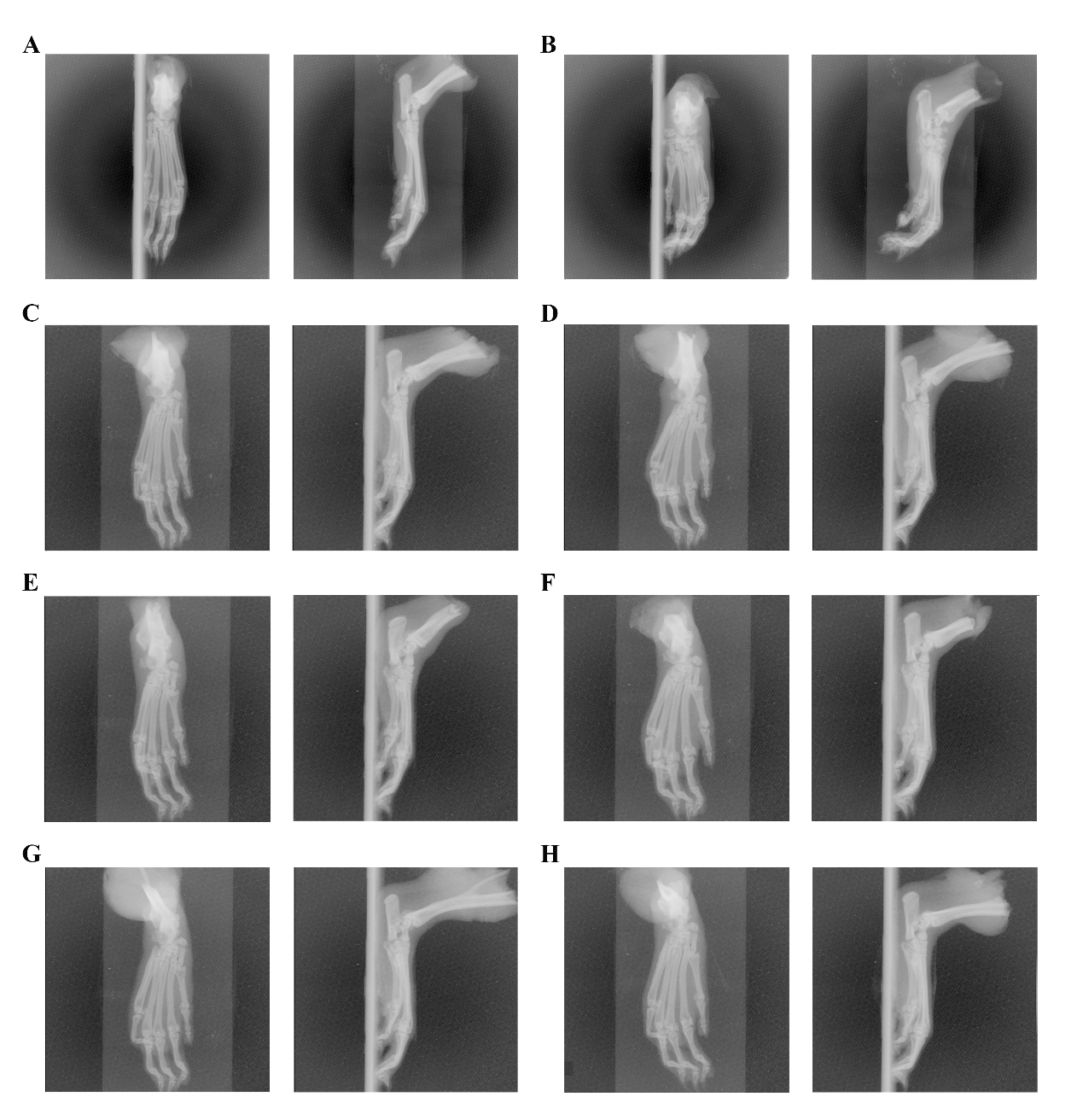


Supplementary Figure 4. X-ray image of AA rat’s joints. The primary side of AA rats’ joints were obtained on day 28 after adjuvant injection. A representative X-ray image was shown: (A) Normal control rats treated with vehicle, (B) AA control rats treated with vehicle, (C) AA rats treated with 1mg/kg FTY720, (D) AA rats treated with 0.5 mg/kg MTX, (E) AA rats treated with 0.3 mg/kg IMMH001, (F) AA rats treated with 0.6 mg/kg IMMH001, (G) AA rats treated with 1.2 mg/kg IMMH001, (H) AA rats treated with 1.2 mg/kg IMMH001.

Supplementary Table 1. Histological score of joints in AA model. (HE staining)

| **Group** | **Dose**  **(mg/kg)** | **Number**  **(n)** | **Histological score**  **(Mean ± SD)** |
| --- | --- | --- | --- |
| Normal Control | - | 6 | 0 ± 0 |
| Arthritic Control | - | 6 | 7.3 ± 1.5 |
| MTX5 | 0.5 | 6 | 5.7 ± 1.0^*^ |
| FTY720 | 1.0 | 6 | 5.5 ± 1.0^*^ |
| IMMH001 | 0.3 | 6 | 7.3 ± 1.5 |
| IMMH001 | 0.6 | 6 | 7.0 ± 1.1 |
| IMMH001 | 1.2 | 6 | 5.5 ± 1.0^**^ |
| IMMH001 | 2.4 | 6 | 5.8 ± 1.2^*^ |

Compared to Arthritic Control, ^*^p﹤0.05, ^**^ p﹤0.01

Supplementary Table 2. Histological score of joints in CIA model. (HE staining)

| **Group** | **Dose**  **(mg/kg)** | **Number**  **(n)** | **Histological score**  **(Mean ± SD)** |
| --- | --- | --- | --- |
| Normal Control | - | 8 | 0 ± 0 |
| Arthritic Control | - | 8 | 10.8 ± 2.0 |
| MTX5 | 0.5 | 8 | 7.1 ± 1.2^**^ |
| FTY720 | 1.0 | 8 | 6.6 ± 1.3^**^ |
| IMMH001 | 0.3 | 8 | 9.9 ± 1.4 |
| IMMH001 | 0.6 | 8 | 7.9 ± 1.2^**^ |
| IMMH001 | 1.2 | 8 | 6.6 ± 1.4^**^ |
| IMMH001 | 2.4 | 8 | 5.6 ± 1.1^**^ |

Compared to Arthritic Control, ^*^p﹤0.05, ^**^ p﹤0.01
